# MiR-218 Induces Neuronal Differentiation of ASCs in a Temporally Sequential Manner with Fibroblast Growth Factor by Regulation of the Wnt Signaling Pathway

**DOI:** 10.1038/srep39427

**Published:** 2017-01-03

**Authors:** Feihu Hu, Bo Sun, Peng Xu, Yanliang Zhu, Xian-Hui Meng, Gao-Jun Teng, Zhong-Dang Xiao

**Affiliations:** 1 State Key Laboratory of Bioelectronics, Southeast University, Nanjing, Jiangsu, China; 2 Medical School, Southeast University, Nanjing, Jiangsu, China; 3 Jiangsu Key Laboratory of Molecular and Functional Imaging, Department of Radiology, Zhongda Hospital, Southeast University, Nanjing, Jiangsu, China

## Abstract

Differentiation of neural lineages from mesenchymal stem cells has raised the hope of generating functional cells as seed cells for nerve tissue engineering. As important gene regulators, microRNAs (miRNAs) have been speculated to play a vital role in accelerating stem cell differentiation and repairing neuron damage. However, miRNA roles in directing differentiation of stem cells in current protocols are underexplored and the mechanisms of miRNAs as regulators of neuronal differentiation remain ambiguous. In this study, we have determined that *miR-218* serves as crucial constituent regulator in neuronal differentiation of adipose stem cells (ASCs) through Wnt signaling pathway based on comprehensive annotation of miRNA sequencing data. Moreover, we have also discovered that miR-218 and Fibroblast Growth Factor-2 (FGF2) modulate neuronal differentiation in a sequential manner. These findings provide additional understanding of the mechanisms regulating stem cell neuronal differentiation as well as a new method for neural lineage differentiation of ASCs.

Mesenchymal stem cells are the ideal candidates for regenerative medicine and tissue engineering^1^. Generating neuronal cells from stem cells is an attractive approach given the limited intrinsic capacity of neurons in repairing neural tissue. *In vitro* studies have shown that the mesenchymal stem cells could differentiate into mature neurons expressing neuronal specific markers after exposure to various chemical agents^2^,^3^,^4^. However, these chemical induction methods are usually of low efficiency and considerable cytotoxicity. Recently, gene therapy has developed to meet this challenge. The approach involves the use of multipotential cells such as bone marrow-derived mesenchymal stem cells (BMSCs), muscle-derived stem cells (MDSCs) and adipose-derived stem cells (ASCs), which are engineered to overexpress factors that are of crucial roles of neurogenesis for promoting neuronal differentiation^5^,^6^,^7^.

Gene expression and related function of stem cell are controlled by a newly discovered class of short 22 nucleotides Micro-RNAs (miRNAs). MiRNAs interact with complex signal transduction pathways, including those involved in neuronal formation and development, by regulating the protein translation of specific cellular mRNAs and mRNAs degradation^8^,^9^,^10^,^11^. In the last decade, there has been an increase in our understanding of the role of miRNAs in neuronal development and stem cell neuronal differentiation, where miRNAs have shown to be involved in important genes that control cell pluripotency. Meanwhile, Researchers endeavor to manipulate the expression of particular miRNAs in order to promote stem cells differentiation into neural progenitor cells or authentic neural cells^12^,^13^,^14^,^15^. For example, *miR-146a* has been shown to be a key regulator of stem cell survival when the cells were incubated with induced factor (Diazoxide) by targeting *Fas* in the NF-κB signaling^16^. *Let-7i* was shown to be a novel and potent inhibitor of neuronal differentiation that targeted *Mash1* and *Ngn1* by participating in Sox2–Lin28 pathway on neurogenic process^17^. *MiR-124* facilitates the maintaince of the neuronal state by targeting the Specificity protein (*Sp1*) which suppressed in differentiated neurons^18^. It has also been reported that the overexpression of *miR-9* promotes neuronal differentiation by targeting the *Tlx1* transcription factor and DNA binding-2 inhibitor^19^. Wang *et al*. have suggested that JMJD1C represses stem cell neuronal differentiation at least partially by epigenetically sustaining *miR-302* expression and thus JMJD1C knockdown is sufficient to trigger neuronal differentiation upon withdrawal of exogenous FGF^20^.

In this study, we have successfully induced neuronal differentiation of ASCs by Retinoic Acid (RA) treatment. To systemically monitor the expression of miRNAs during ASCs differentiation, we determined the miRNA expression profile of ASCs, incubated with or without RA by high-throughput deep sequencing using Applied Biosystems SOLiD System. After comprehensive analysis of miRNAs sequencing data (miRNA profiling), we found that *miR-218* is specifically expressed in neuronal differentiation. Furthermore, our study highlighted an intricate gene regulatory network and pathway (Wnt signaling) which is in turn highly related with *miR-218* expression. Consequently, our results showed that the interaction of *miR-218* and Wnt signaling had a crucial role which efficiently facilitate the differentiation of ASCs. After confirming that the expression of *miR-218* alone was not enough to differentiate ASCs into neuronal cells, we demonstrated that *miR-218* and Fibroblast Growth Factor-2 (FGF2) together regulate the generation of neuronal cells from ASCs in a temporally sequential manner. Our study, for the first time has provided a new insight into the time-sequential regulation mechanisms of neuronal differentiation.

## Results

### MiRNA expression profile analysis revealed that the Wnt signaling pathway and *miR-218* were crucial for neuronal differentiation of ASCs

**Figure 1:**
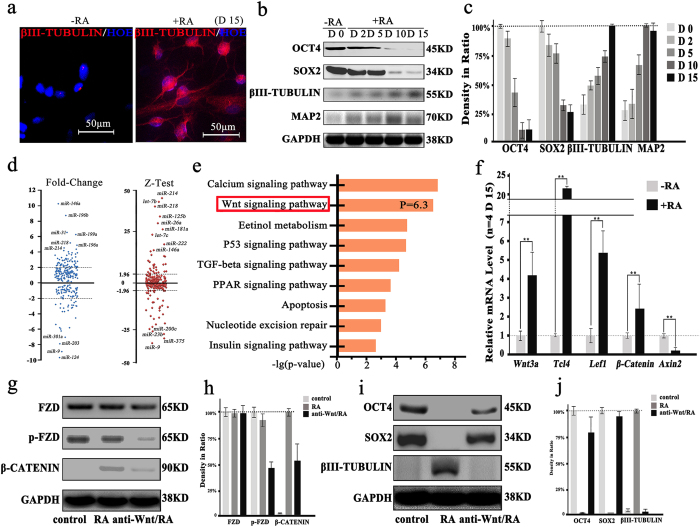
MiRNAs expression analysis in neuronally differentiated adipose stem cells (ASCs) using the DAVID database and effects of Wnt signaling pathway inhibition by ICG-001 assessed by immunofluorescence, Western blotting and qRT-PCR. (**a**) Immunofluorescence photomicrograph image of βIII-TUBULIN (red) and HOECHST (HOE, blue) staining in ASCs of non-treated cells (-RA group) and RA-treated cells (+RA group) for 15 days (D 15). (**b**) Western blotting of OCT4, SOX2, βIII-TUBULIN and MAP2 in the –RA and +RA groups at different time points and GAPDH is used as control. (**c**) Proteins expression levels, quantified by determining the gray value. (**d**) The expression ratios and evaluation, based on the mathematical model (using Fold-change and Z-test method), for all-known miRNAs that are detected between -RA and +RA groups. (**e**) Top KEGG pathways are summed up from the DAVID database based on *P-value*. (**f**) Expressions of key gene (*Wnt3a, Tcf4, Lef1, β-Catenin* and *Axin2*) in Wnt signaling pathway are detected by qRT-PCR between -RA and +RA groups (** p < 0.01, n = 4). (**g**) Western blotting analysis of Wnt signaling pathway marker proteins (FZD and phosphorylated FZD in the cytoplasm and the β-CATENIN in the nuclear) in control group (non-treated), RA group (RA-treated for 15 days) and anti-Wnt/RA group (pre-treated by the inhibitor of Wnt signaling pathway, ICG-001 and the group is incubated with RA for 15 days), GAPDH is used as control. (**h**) The proteins expression levels, quantified by determining the gray values (n = 2). (**i**) Western blotting analysis of the stem cell stemness marker (OCT4 and SOX2) and neural cell marker (βIII-TUBULIN) expression in control group, RA group and anti-Wnt/RA group and GAPDH is used as control. (**j**) Proteins expression levels, quantified by determining the gray value (n = 2).

After 15 days of incubation with RA, the neurite outgrowth has been observed when the ASCs is differentiated into the neuronal lineage. The differentiation of ASCs into the neuronal lineage is confirmed by the expression of the neural terminal differentiation marker, *βIII-Tubulin*, using immunofluorescence (Fig. 1a). The protein expression of differentiation markers such as OCT4, SOX2, βIII-TUBULIN and MAP2 are also monitored at different time points (Day 0, Day 2, Day 5, Day 10 and Day 15) of RA treatment. (Figure 1b,c). The reduction of stemness markers (OCT4 and SOX2) is accompanied by enhancing the neural cell markers (βIII-TUBULIN and MAP2). The percentage of cells quantifies for this transformation (see Supplementary Figs S1 and S2). To elucidate the expression pattern of miRNAs during neuronal differentiation, high-throughput deep sequencing is performed using an Applied Biosystems SOLiD System. From the miRNA profiling results, about dysregulated 654 miRNAs are summarized. With the fold-change and Z-test analysis in the sequencing results, we have found that the expression levels of miRNAs are widely affected while the ASCs are differentiated into neuronal lineage and some miRNAs expression levels are more tempestuously regulated, including *miR-146a, miR-196b, miR-31, miR-218, miR-214, miR-203, miR-124, miR-26a, miR-222, miR-375, miR-9*, and *let-7* family (Fig. 1d). The expression levels of some miRNAs are implicated in the development of neurons, such as *miR-9, miR-214* and the *let-7* family (the expression levels of *miR-9, miR-146a* and *miR-214* are detected at 3 time points, see supplementary Fig. S3). Meanwhile, we evaluate the target genes of this miRNA pool by bioinformatics and subject to DAVID database. The functions of the target genes predicted by obviously altering miRNAs are annotated with KEGG signaling pathway analysis. From the *P-Value* analysis in the terms of the biological process, the Wnt signaling pathway (*P - Value* = 6.3) is likely to be critical for ASCs neuronal differentiation (Fig. 1e). The key gene expressions in Wnt signaling pathway (*Wnt3a, Tcl4, Lef1, β-Catenin* and *Axin2* in Wnt/β-Catenin pathway) predicted with bioinformatics are validated by qRT-PCR (Fig. 1f). After addition of ICG-001 protein and subsequently adding RA for 15 days (anti-Wnt/RA group), Wnt signaling pathway is effectively inhibited by decreasing the expression levels of phosphorylation FZD (p-FZD) and β-CATENIN (Fig. 1g,h). As expected, OCT4 and SOX2 protein levels are unchanged and βIII-TUBULIN is undetectable in anti-Wnt/RA group (Fig. 1i,j).

**Figure 2:**
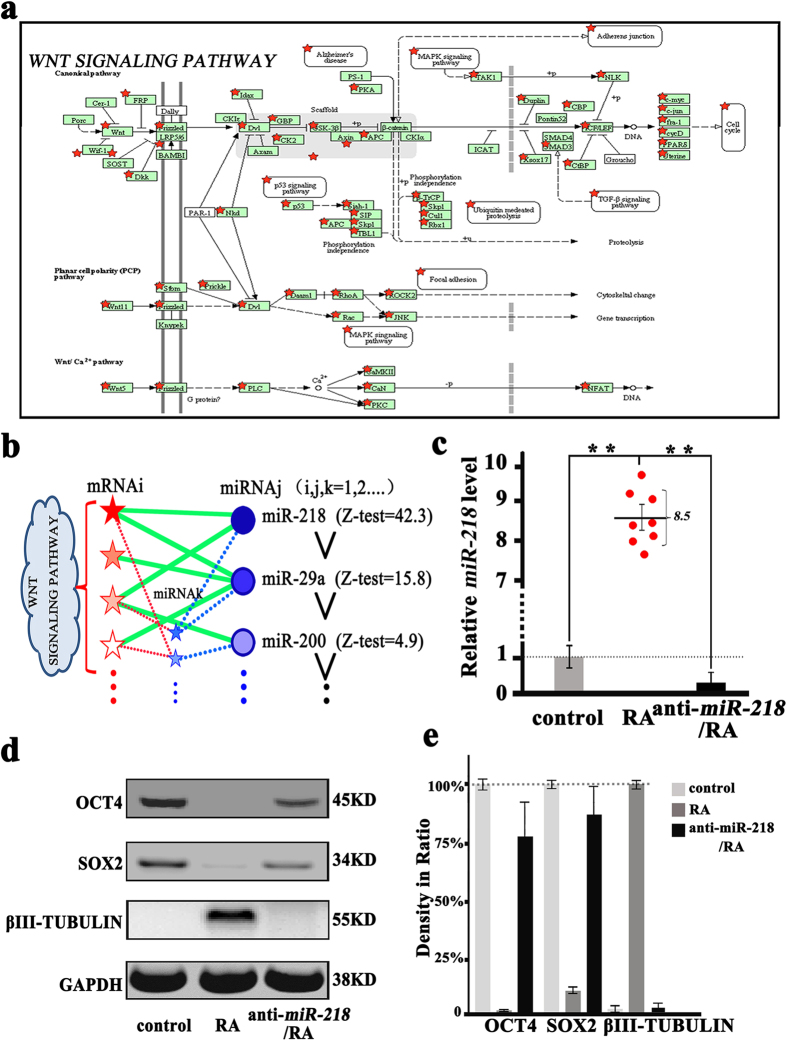
MiR-218 expression and its regulation of the Wnt signaling pathway assessed by the Western blotting and qRT-PCR. (**a**) In the +RA group (RA-treated group), based on the database prediction (TargetScan version 6.2 database) and the KEGG analysis, the genes involved in the Wnt signaling pathway (frame fill with green) and several key genes (red pentacles) in the Wnt signaling pathway. (**b**) Diagram showing the link between the key miRNAs and its target genes which are involved in or targeted on the regulation of Wnt signaling pathway. The genes included in Wnt signaling pathway (red pentacles) and regulated Wnt signaling (blue pentacles) are targeted by miRNAs (blue circles) and the key miRNAs (*miR-218, miR-29a* and *miR-200 et al.*) are summarized according the Z-test ranking results. *MiR-218* (Z-test = 42.3) is found to be in the front rank. (**c**) Relative *miR-218* expression for the control group (RA-non-treated cells), RA group (RA-treated for 15 days) and anti-*miR-218*/RA group (ASCs are transfected by anti-*miR-218* plasmids for 48 h and RA treated for 15 days) are determined by qRT-PCR (**p < 0.01, n = 8). (**d**) Western blotting analysis of OCT4, SOX2 and βIII-TUBULIN expression in the control group, RA group and anti-*miR-218*/RA group and GAPDH is used as control. (**e**) Protein expression levels, quantified by determining the gray value (n = 3).

We further investigate the Wnt signaling pathway genes. From KEGG analysis results, the red pentacles reveal the key genes closely related to the ASCs neuronal differentiation process (Fig. 2a). The genes involving in Wnt signaling (red pentacles) or regulation of Wnt signaling (blue pentacles) are targeted by miRNAs (blue circles). From our sequencing and predicted data, the top level among the differentially expressed miRNAs is highlighted and reveals that *miR-218* has significantly up-regulation after RA treatment (Z-test = 42.3, Fig. 2b), which is accord with the previous reports about the key regulator in Wnt Signaling^21^,^22^,^23^. Therefore, *miR-218* is considered to be crucial for ASCs neuronal differentiation. Indeed, we find RA supplementation in culture medium increases the endogenous *miR-218* expression by almost 8.5-fold (Fig. 2c) and downregulates the expression of the OCT4 and SOX2 simultaneously (Fig. 2d,e). However, after anti-*miR-218* transfection, subsequent RA treatment does not enhance βIII-TUBULIN expression (in anti-*miR-218*/RA group, Fig. 2d,e).

Taken together, these data demonstrated that the Wnt signaling pathway and *miR-218* both participate and positively promote ASCs neuronal differentiation.

### MiR-218 regulates Wnt signaling pathways but is insufficient to induce ASCs differentiation into neural cells

**Figure 3:**
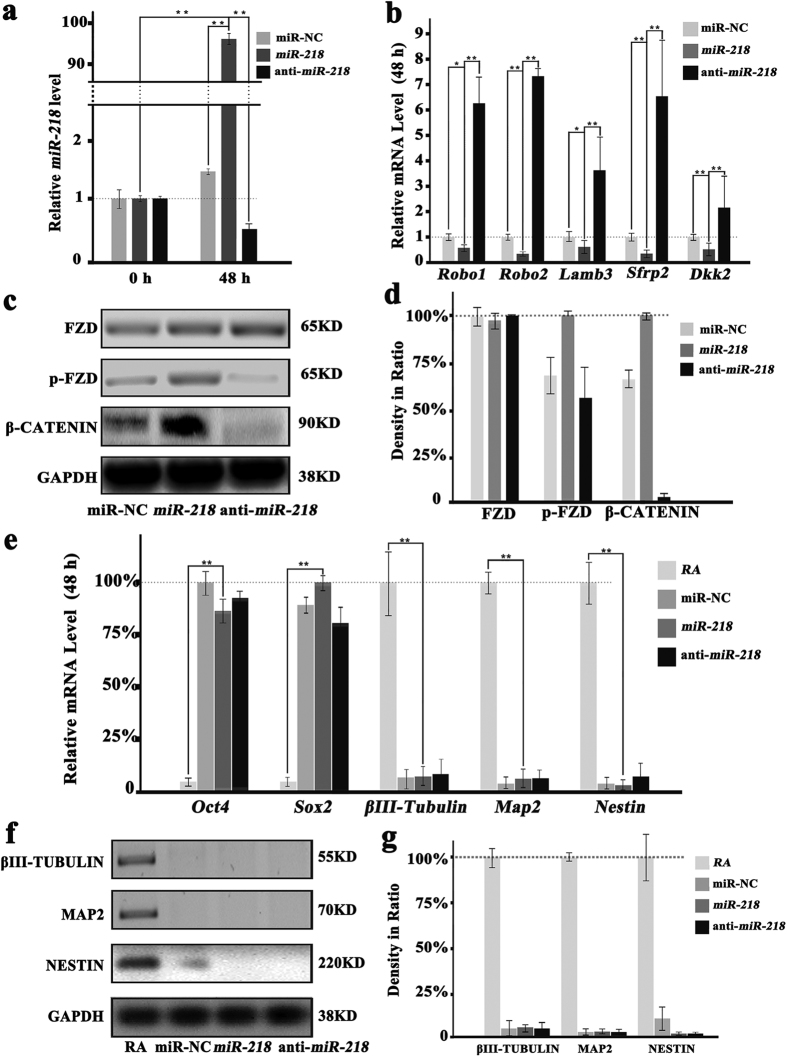
Expression of miR-218 and of components of the Wnt signaling pathway on ASCs neuronal differentiation, monitored by Western Blotting and qRT-PCR. (**a**) The relative *miR-218* expression levels for ASCs transfected with control plasmid (miR-NC group), *miR-218* plasmid (*miR-218* group) and anti-*miR-218* plasmid (anti-*miR-218* group) after 48 h are detected by qRT-PCR (**p < 0.01, n = 4). (**b**) The qRT-PCR results of some *miR-218* target (*Robo1, Robo2, Lamb3, Sfrp2* and *Dkk2*) mRNAs expression, detected in miR-NC group, *miR-218* group and anti-*miR-218* group. (**c**) Western blotting results of Wnt signaling pathway markers (FZD and p-FZD in the cytoplasm and β-CATENIN in the nuclear) in the miR-NC group, *miR-218* group and anti-*miR-218* group and GAPDH is used as control. (**d**) Proteins expression levels, quantified by determining the gray value (n = 2). (**e**) The qRT-PCR results of *Oct4, Sox2, βIII-Tubulin, Map2 and Nestin* mRNA expression levels in the RA-treated group (RA group) and three plasmid treated groups (miR-NC group, *miR-218* group and anti-*miR-218* group). (**f**) Western blotting results of βIII-TUBULIN, MAP2 and NESTIN in the four groups (RA group, miR-NC group, *miR-218* group and anti-*miR-218* group) and GAPDH is used as control. (**g**) Protein expression level, quantified by determining the gray value (n = 2).

*MiR-218* is specifically active in developing motor neurons. The robust upregulation of *miR-218* in ASCs, differentiate ASCs into the neural lineage inspires us to investigate whether over expressing *miR-218* may induce the neuronal differentiation of ASCs through Wnt signaling pathway. The *miR-218* transfection markedly increases the endogenous *miR-218* levels by almost 100-fold (Fig. 3a). While the transfection of anti-*miR-218* significantly decrease the *miR-218* expression. The expression of *miR-218* target genes like *Robo1, Robo2* and *Lamb3* and the Wnt signaling pathway antagonist genes such as *Sfrp2* and *Dkk2* are validated by qRT-PCR with the cells transfected with *miR-218* and anti-*miR-218* (Fig. 3b). In comparison to the transfection of *anti-miR-218* and the controls (miR-NC), transfection of *miR-218* dramatically enhances p-FZD levels and elevates nuclear accumulation of β-CATENIN (Fig. 3c,d). These results indicate that *miR-218* transfection can activate Wnt signaling pathway. Meanwhile, it seems that the anti-Wnt does not have any effect on the expression of *miR-218* (see Supplementary Fig. S4).

However, both the mRNAs levels of *Oct4, Sox2, βIII-Tubulin, Map2* and *Nestin* (Fig. 3e) and the protein expression studies (Fig. 3f,g) prove that, overexpression of *miR-218* alone cannot induce *βIII-Tubulin, Map2* and *Nestin* expression in the absence of RA. These results confirm that, although *miR-218* positively regulates Wnt signaling pathway, which alone is insufficient to induce ASCs differentiation into neural cells.

### FGF2 and miR-218 co-operate sequentially in ASCs neural differentiation

**Figure 4:**
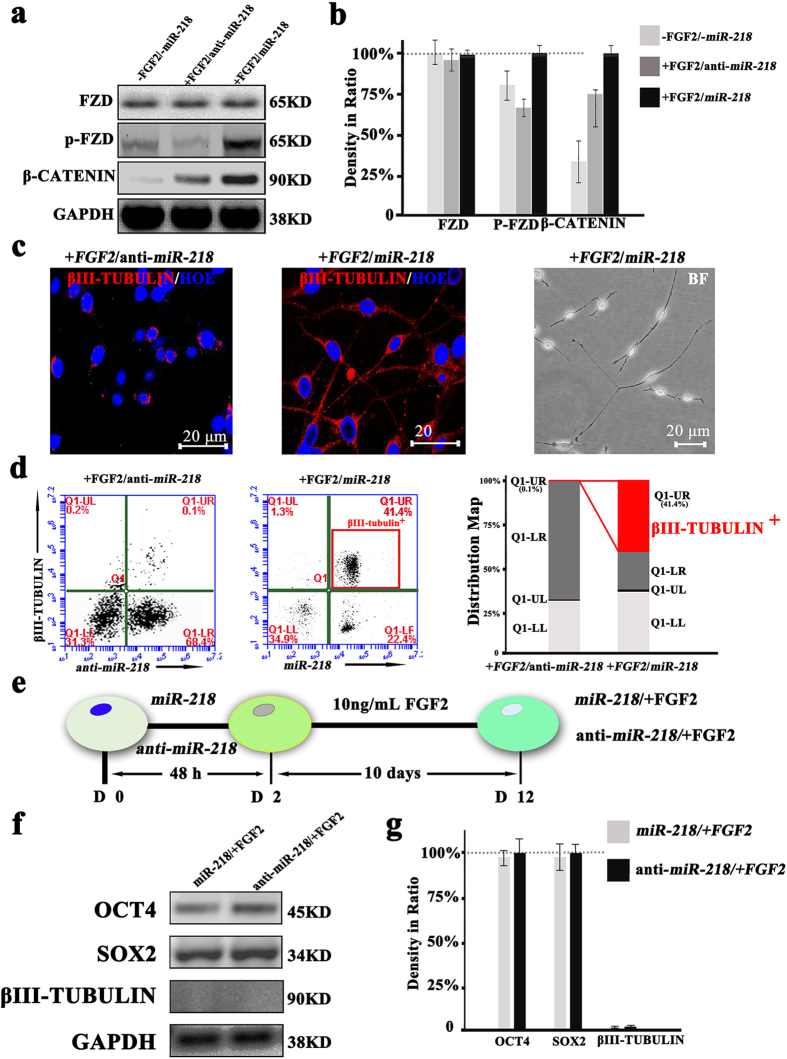
Co-ordinated effect of FGF2 and miR-218 on neural differentiation of ASCs assessed by Western blotting, flow cytometry, and cell imaging following immunofluorescence staining. (**a**) Western blotting analysis of three markers of Wnt signaling pathway in –FGF2/-*miR-218* group (without any FGF2 or plasmid treated), +FGF2/anti-*miR-218* group (ASCs were incubated with 10 ng/mL FGF2 for 10 days and transfected with anti-*miR-218* plasmid for 48 h), +FGF2/*miR-218* group (ASCs were incubated with 10 ng/mL FGF2 for 10 days and transfected with *miR-218* plasmid for 48 h) and GAPDH is used as control. (**b**) Proteins expression levels, quantified by determining the gray value (n = 3). (**c**) Immunofluorescence image of βIII-TUBULIN (red) and HOE (blue) staining in two groups (+FGF2/anti-*miR-218* group and +FGF2/*miR-218* group). Photomicrograph image of ASCs in +FGF2/*miR-218* group. Scale bars, 20 μm. (**d**) The ASCs neuronal differentiation (βIII-TUBULIN positive expression in the +FGF2/anti-*miR-218* group and +FGF2/*miR-218* group) are performed by two-color flow cytometric analysis. The dot blot and data table (the red frame in the quadrants and red section in the column diagram) shows the proportion of the βIII-TUBULIN positive expression in +FGF2/anti-*miR-218* group and +FGF2/*miR-218* group. (**e**) Schematic illustration and timelines of ASCs cultured with FGF2 and *miR-218* shows the sequential regulation method. ASCs are transfected with *miR-218* plasmid. After 48 h (D 2), cells are maintained in medium supplemented with 10 ng/mL FGF2 for 10 days (D 12) (*miR-218*/+FGF2 group). (**f**) Western blotting analysis for OCT4, SOX2 and βIII-TUBULIN protein in *miR-218*/+FGF2, anti-*miR-218*/+FGF2 and GAPDH is used as control. (**g**) Proteins expression levels, quantified by determining the gray value (n = 2).

Previous studies demonstrate that the FGF signaling pathway participates in neurogenesis and central nervous system formation^24^,^25^,^26^. In our earlier studies, we identified that addition of FGF2 (10 ng/mL) may work as a pre-induction factor and affect ASCs neuronal differentiation^27^. Herein, we speculate that FGF2 may interact with *miR-218* to induce neuronal differentiation. To confirm this, we pre-treat ASCs with FGF2 (10 ng/mL) for 10 days followed by transfecting with *miR-218*. Subsequently, elevation of *miR-218* levels in ASCs (+FGF2/*miR-218* group) increases the expression of Wnt signaling pathway markers (p-FZD and β-CATENIN) in the +FGF2/*miR-218* group compared to the -FGF2/-*miR-218* and +FGF2/anti-*miR-218* groups (Fig. 4a,b). This indicates, FGF2 and *miR-218* work synergistically for enhancing Wnt signaling pathway. The morphological transformation to neural-like cells and the expression of *βIII-Tubulin* are confirmed by immunofluorescent imaging and photomicrograph (Fig. 4c). At the same time, the two-color Flow Cytometry (flow cytometric dot plots) shows, there is an increase (0.1% to 41.4%) of double-positive (*βIII-Tubulin* + cells) cells in +FGF2/*miR-218* group compared to +FGF2/anti-*miR-218* group (Fig. 4d). These results indicate, the temporal relationship between FGF2 and *miR-218* on the neuronal differentiation. The FGF2 pretreatment cooperatively interacts with *miR-218* to induce ASCs into neural lineage.

To evaluate in sequential manner, we treat cells with FGF2 followed by transfecting with *miR-218 (miR-218*/+FGF2 group) or anti-*miR-218* (anti-*miR-218*/+FGF2 group) (Fig. 4e). Interestingly, neither morphological changes nor βIII-TUBULIN expression can be detected (Fig. 4f,g) in the above treated cells. These results indicate that pre-induction with FGF2 is necessary to facilitate the neuronal induction effect of *miR-218* in ASCs. In addition, we find that *miR-218* and FGF2 does not form the negative feedback loop (see Supplementary Fig. S5).

Taken together, our data indicate, elevation of FGF2 and *miR-218* cooperatively induces stem cells to differentiate in a temporally sequential manner *via* the Wnt signaling pathway.

## Discussion

Micro-RNAs inhibit translation and mediate mRNA decay through sequence-specific base pairings with specific region of target genes. Researchers endeavor to manipulate the expression of particular miRNAs in order to promote stem cells differentiation into neural progenitor cells or authentic neural cells^28^,^29^,^30^. In this study, we analyzed miRNA expression profiling that significantly changed when ASCs were treated with RA for neuronal differentiation. We identified that certain miRNAs exhibited tremendous changes in their expression during differentiation (including *let-7* family, *miR-146a, miR-196b, miR-218, miR-214, miR-203, miR-124, miR-26a, miR-222, miR-375, miR-9*), which have been shown to facilitate neurogenesis^15^,^31^,^32^,^33^,^34^,^35^,^36^.

Similarly, we predicted from bioinformatics analysis^37^,^38^,^39^, and then found, that Wnt signaling pathway was closely involved and played a pivotal role in the neural differentiation process of ASCs. In the Wnt signaling pathway (involving Fzd/β-Catenin pathway), the combination of Wnt proteins and the receptors led to an increase in activity of glycogen synthase kinase 3β (*Gsk3β*) and *Axin2*. Then, the *β-Catenin* undergoes a nuclear translocation where it accumulated and formed complexes with transcription factors, activating a number of intracellular signaling pathways^40^,^41^,^42^. Previous evidence revealed that Wnt signaling pathway promoted stem cell self-renewal and participated in neurogenesis^43^. Wiggan *et al*. hypothesized that, during early placode development, *Pax3* and *Sox2* activated Wnt signaling pathway through the enhancer *N-1c*^44^. Their research revealed that, the direct involvement of Wnt signaling in the initiation of neural plate development. Studies by Elizalde *et al*. indicated that *Wnt-4* and *Wnt-11* (Wnt signaling pathway regulator) promoted early neuronal differentiation by diminishing the RA-induced downregulation of *Oct4* and *Nanog* and the upregulation of *Pax6, Ascl1, Hoxc5*, and *Neurod1*^45^.

Furthermore, certain miRNAs have shown to be involved with important genes that controlled the cell pluripotency and mediated the induction of pluripotent stem cells by targeting the Wnt signaling pathway. For example, considerable evidence suggested that the Wnt signaling pathway has been regulated by *miR-499, miR-355, miR-375, miR-27, miR-29, miR-17, miR-142* and *miR-218*^46^,^47^,^48^,^49^,^50^,^51^,^52^,^53^,^54^. The *Dkk2, Sfrp2* and *Sost* are reported as the Wnt signaling pathway inhibitors and *miR-218* targets these inhibitors as a positive feedback loop with Wnt signaling pathway^23^,^55^. Based on the sequencing data, we selected *miR-218* for further investigation, not only because of its extreme differential expression in comparison to other miRNAs (Fig. 1d) but mainly of its involvement in the Wnt signaling pathway (Fig. 2b–d).

To further support the hypothesis that Wnt signaling pathway and *miR-218* were closely involved in the process of ASCs neural differentiation, we used anti-*miR-218* and the inhibitor ICG-001 to block ASCs neuronal differentiation and supplemented the ASCs culture medium with RA. Our experimental results verified the predictive identification of *miR-218* and Wnt signaling in neural differentiation and using our modified bioinformatics analysis method further demonstrated their function as key cellular triggers of neuronal differentiation. Interestingly, *miR-218* did not appear to play an instructive role neither in mesenchymal stem cell fate determination nor in motor neuron fate determination on its own. Given that the overexpression of *miR-218* alone was not sufficient to induce the formation of motor neurons in chick neural tube or mouse embryonic stem cells (ESCs) and to induce the neuronal differentiation of ASCs as well^56^,^57^. Therefore, we speculated that there might exist a method of combinative regulation in the ASCs neuronal differentiation.

FGF2 belongs to the family of heparin-binding growth factors and has been described as a mitotic activator in the stem cells differentiations^27^,^58^. The FGF2 treatment of cultured stem cells provided mitogenic support and predominated in the induction. In the present study, we included FGF2 in the culture medium as a pre-induction factor for neural differentiation. With this pre-induction and the overexpression of *miR-218*, Wnt signaling pathway was stimulated to a greater extent compare to other conditions such as +FGF2/anti-*miR-218* or *miR-218*. Additionally, we demonstrated that, synergistically, the supplementation of FGF2 and overexpression of *miR-218* prompted the differentiation of ASCs into neural cells (Fig. 4c,d).

A number of studies have investigated the effects of various inductive factors at different stages of differentiation in *in-vitro*. For instance, Tang *et al*. revealed that appropriate timing of *Wnt-1* expression was necessary for the appropriate RA-induced expression of the neural phenotype in P19 cells^59^. Handorf *et al*. delineated two stages of chondrogenesis and identified that developmental days 9–12 represented an important regulatory point in the chondrogenic program of hMSCs by examining the sequential administration of TGFB1, BMP7, and IGF1 within specific temporal windows^60^. Similarly, the major principle of stem cells neuronal differentiation may have a multi-step process, and that cells exhibit spatial and temporal responses to signaling pathways and their regulators (i.e., miRNAs). Therefore, we classified mesenchymal stem cells neural differentiation could be divided into two sequential stages: “induction” and “differentiation”. The delivery of FGF2 was used for the induction of differentiation in the initial step, during which ASCs displayed a stage of increased activity triggered by FGF2 and were “conditioned” for the subsequent elevation of *miR-218*. Finally, we validated the sequential link between bFGF (FGF2) and *miR-218* during ASC neural differentiation. Further investigations of the molecular mechanisms underpinned this link and the synergistic effects of FGF2 and *miR-218* on the Wnt signaling pathway during neuronal differentiation were warranted.

In this study, we identified that Wnt signaling pathway and *miR-218* were closely related to ASCs neuronal differentiation. We also demonstrated that *miR-218* overexpression alone failed to induce ASCs neuronal differentiation and FGF2 pretreatment cooperatively interacted with *miR-218* to generate neural cells. Furthermore, FGF2 and *miR-218* were shown to operate in a temporally sequential manner to promote the differentiation of ASCs into the neural lineage. Our results augmented, the current understanding of the developmental processes of neural differentiation and provided important insights into how miRNAs contributed to this process, which could assist the development of novel inductive approaches for neural tissue regeneration.

## Materials and Methods

### ASCs isolation and differentiation

ASCs were obtained from four-week old female Sprague-Dawley rats (weight 100–130 g), as previously described^61^. The use of all animal samples were approved by and carried out in accordance to the medical ethics committee of Southeast University, China. ASCs were digested and seeded. The cells were cultured in basal medium composed of Dulbecco’s modified Eagle’s medium (DMEM; Thermo Fisher Scientific, USA), 5% fetal bovine serum (FBS; Gibco Lab., USA), 1% penicillin/streptomycin (Thermo Fisher Scientific) with or without FGF2 (Peprotech, USA). The medium was replaced every 3 days for a period of 10 days. ASCs were then seeded into 12-well plates and neuronal differentiation (RA-treated, +RA group) was performed over a period of 15 days using basal medium supplemented with 100 ng/mL Retinoic Acid (RA; Sigma Aldrich, USA). The non-treated group (control, −RA group) was cultured contemporaneously.

### MiRNA analysis

Total RNA were extracted from RA-treated and non-treated cells and altered miRNAs expression were detected by sequencing of the Applied Biosystems SOLiD System, as previously described^37^,^62^,^63^. Altered expression of miRNAs and prediction of effects on their target genes were analyzed following the method (based on the Z-test calculation method, a mathematical model to evaluate the comprehensive repression rate of specific mRNAs using total miRNA expression profiling) reported previously^37^,^64^,^65^. Briefly, the target genes levels between RA-treated (+RA group) and non-treated (−RA group) cells were predicted by the tools of TargetScan version 6.2 database. The identified lists of genes were subjected to functional annotation, clustering and analysis using the KEGG analysis based on the Database for Annotation, Visualization and Integrated Discovery (DAVID) Bioinformatics Database (https://david.ncifcrf.gov/).

### Quantitative real-time PCR

The miRNAs and mRNAs expression were measured by quantitative Real-Time PCR (qRT-PCR) using ABI 7500 System (Life Technologies, USA) and standard protocols (All the Primers are listed in Supplementary Table S1 online). Relative amounts were determined using the 2^−ΔΔCt^ method.

### ICG-001 treatment

ICG-001 protein inhibits the Wnt signaling pathway by binding to the element-binding protein (CBP)^66^. ASCs were seeded into 12-well plates and maintained at CO2 incubator. After 24 h of seeding, the final concentrations of 5 μM ICG-001 (Selleckchem, USA) was added into the ASCs medium for 2 days, followed by 100 ng/mL of RA was added and further cultured for 15 days (anti-Wnt/RA group).

### Western blotting

Total cellular proteins and nuclear proteins were separately extracted using the Total Protein Isolation Kit (Sangon Company, China) and Nuclear Protein Isolation Kit (Sangon Company). Proteins were quantified by Bicinchoninic acid assay kit (Sangon Company). The protein samples were loaded on SDS–PAGE gels and electrophoresed under standard conditions. Western blotting was performed using nitrocellulose membranes. After blocking, membranes were incubated with primary antibodies (1:200–1:500) at 4 °C overnight. After rinsing, incubation was conducted with secondary horseradish peroxidase-conjugated goat anti-rabbit or mouse IgG antibody (Bioss Biotechnology, China) and exposed to film. Primary antibodies included anti-Frizzled (FZD, Immunoway, Catalog: YT1783, USA), anti-phospho-Frizzled (p-FZD, Immunoway, Catalog: YP0173), anti-β-Catenin (Boster Biotechnology, Catalog: BM1575, China), anti-Oct4 (Santa Cruz Biotechnology, Catalog: sc-5279, USA), anti-Sox2 (Santa Cruz Biotechnology, Catalog: sc-17320), anti-βIII-Tubulin (Santa Cruz Biotechnology, Catalog: sc-58888), anti-Map2 (Santa Cruz Biotechnology, Catalog: sc-20172), anti-Nestin (Santa Cruz Biotechnology, Catalog: sc-20978), and anti-Gapdh (Cell Signaling Technology, Catalog: #2118, USA).

### MiRNA transfection

ASCs were seeded into 12-well plates, after 24h of seeding, 5 μL Superfectin Transfection Reagent (Qiagen, Canada) was added per well, according to the manufacturer’s instructions. Transfections were performed using 20 nM of *miR-NC*, anti-*miR-218* or *miR-218*, respectively. The miRNA plasmids were designed and enhanced green fluorescence protein (eGFP) was used as a reporter gene. After 48 h, transfected cells and transfection efficiency was measured and assayed by detection of eGFP. In the anti-*miR-218*/RA group, the anti-*miR-218* plasmids were transfected for 2 days and the ASCs medium was supplemented with 100 ng/mL RA for 13 days. The βIII-tubulin positive expressions were analyzed by Flow Cytometry using C-Flow software.

### FGF2 pretreatment

ASCs medium was supplemented with 10 ng/mL FGF2 for 10 days, followed by transfected with miR-NC, anti-*miR-218* or *miR-218* plasmids for 2 days. In order to evaluate the temporal relationship, the chronological sequence was exchanged.

### Flow cytometry

Cells were trypsinized with 0.25% trypsin solution (Sigma Aldrich) and fixed with 4% formaldehyde (Sangon Company) in PBS at 37 °C for 0.5 h. After rinsing, the cells were incubated with rabbit anti-rat βIII-Tubulin primary antibody (1:200 in 1% BSA; Santa Cruz Biotechnology) at 37 °C for 4 h then incubated with the appropriate amount of the secondary antibody, goat anti-rabbit Alexa-Fluor 647 (1:500 in 1% BSA; Invitrogen, USA) for 1 h at 37 °C. During the whole experiment the cells were protected from light. Finally, the cell samples were subjected to Flow Cytometry and corresponding data were analyzed by C-Flow software.

### Immunofluorescent analysis

After induction of RA and/or *miR-218* for a period of time, ASCs were subjected to immunofluorescent staining by rabbit anti-rat βIII-Tubulin primary antibody to detect differentiated neural cells. After washing with PBS, the samples were incubated with the appropriate secondary antibody, mouse anti-rabbit Alexa-Fluor 647 (1:200 in 1% BSA; Cell Signaling Technology) in the dark. Then samples were washed twice with PBS, the nuclei stained with 10 μg/mL Hoechst 33342 (HOE, Sigma Aldrich,) for 0.5 h, and images were obtained using a Revolution XD confocal laser scanning microscope (Andor, Belfast, Northern Ireland).

### Statistical analysis

All data were expressed as mean ± SD. Differences were compared using the Student’s t-test; p-values < 0.05 were considered statistically significant (*p < 0.05, **p < 0.01).

## Additional Information

**How to cite this article**: Hu, F. *et al*. MiR-218 Induces Neuronal Differentiation of ASCs in a Temporally Sequential Manner with Fibroblast Growth Factor by Regulation of the Wnt Signaling Pathway. *Sci. Rep.* **7**, 39427; doi: 10.1038/srep39427 (2017).

**Publisher's note:** Springer Nature remains neutral with regard to jurisdictional claims in published maps and institutional affiliations.

## Supplementary Material

Supplementary Dataset
