# Supplementary material for: MiR-218 Induces Neuronal Differentiation of ASCs in a Temporally Sequential Manner with Fibroblast Growth Factor by Regulation of the Wnt Signaling Pathway
Source: Sci Rep. 2017 Jan 3;7:39427. doi: 10.1038/srep39427 (PMC5206743; doi:10.1038/srep39427)

Supplementary materials for

**MiR-218 Induces Neuronal Differentiation of ASCs in a Temporally Sequential Manner with Fibroblast Growth Factor by Regulation of the Wnt Signaling Pathway**

Feihu Hu, Bo Sun, Peng Xu, Yanliang Zhu, Xian-Hui Meng, Gao-Jun Teng and Zhong-Dang Xiao

**This file includes:**

Supplementary Table. S 1

Supplementary Figs. S 1 to S 6

Supplementary Table. S 1

List of Primers Used for qRT-PCR

| Genes | Forward Primers (5’-3’) | Reverse Primers (3’-5’) |
| --- | --- | --- |
| *Gapdh* | CGATCCCGCTAACATCAAAT | GGATGCAGGGATGATGTTCT |
| *Wnt3a* | ATTGAATTTGGAGGAATGGT | CTTGAAGTACGTGTAACGTG |
| *Tcl4* | GCCTCTCATCACGTACAGCA | GGATGGGGGATTTGTCCTAC |
| *Lef1* | AGCCTGTTTATCCCATCACG | TGAGGCTTCACGTGCATTAG |
| *β-Catenin* | TCCTCCCTCACAGACTCAT | GCTGGGACACGATAATACA |
| *Axin2* | TGACTCTCCTTCCAGATCCCA | TGCCCACACTAGGCTGACA |
| *Robo1* | GGAGGAAAGATGACGGAGAGC | AGATGTTGGGGTTGCTCCTGA |
| *Robo2* | AAGAAAGAGTTAAGGTGGGTGG | TCTGAAGGACCATCAGGTCC |
| *Lamb3* | CCTGTGACCGACTGAC | ACTACATTGGGCAGACAC |
| *Sfrp2* | ATCCTGGAGACAAAGAGCAAGACC | TGACCAGATACGGAGCGTTGATG |
| *Dkk2* | GCCAAACTCAACTCCATCAAGTCC | TCTTACTGCCGCCGAAAGCC |
| *Oct4* | AGGCAGGAGCACGAGTGGA | CGAAGCGGCAGATGGTTGT |
| *Sox2* | CAGGGAGTTCGCAAAAGTCT | AAACCCAGCAAGAACCCTTT |
| *βIII-Tubulin* | TGGAGGTGGCTACATACAGG | TGGGAGGATAGCAGAAGAAC |
| *Map2* | AATTGCCTTCCTCATTCGC | TGTCTTCCAGGTTGGTACCG |
| *Nestin* | TGGAGGTGGCTACATACAGG | TGGGAGGATAGCAGAAGAAC |
| *Fgf2* | GCAGCATCACTTCGCTTCC | TGGAAGAAACAGTATGGCCTTCTG |
| *miR-9* | ACACTCCAGCTGGGTCTTTGGTTATC | TGGTGTCGTGGAGTCG |
| *miR-146a* | ACACTCCAGCTGGGTGAGAACTGAAT | TGGTGTCGTGGAGTCG |
| *miR-214* | ACACTCCAGCTGGGAGAGTTGTCA | TGGTGTCGTGGAGTCG |
| *miR-218* | ACACTCCAGCTGGGTTGTGCTTGATC | TGGTGTCGTGGAGTCG |
| *U6* | GCTTCGGCAGCACATATACTAAAAT | CGCTTCACGAATTTGCGTGTCAT |

Supplementary Fig. S 1

The percentage of ASCs neuronal differentiation in the RA group (the cells are incubated with RA for 15 days, Fig. S 1a) and in the FGF2/ *miR-218* group are estimated by Flow cytometric analysis.


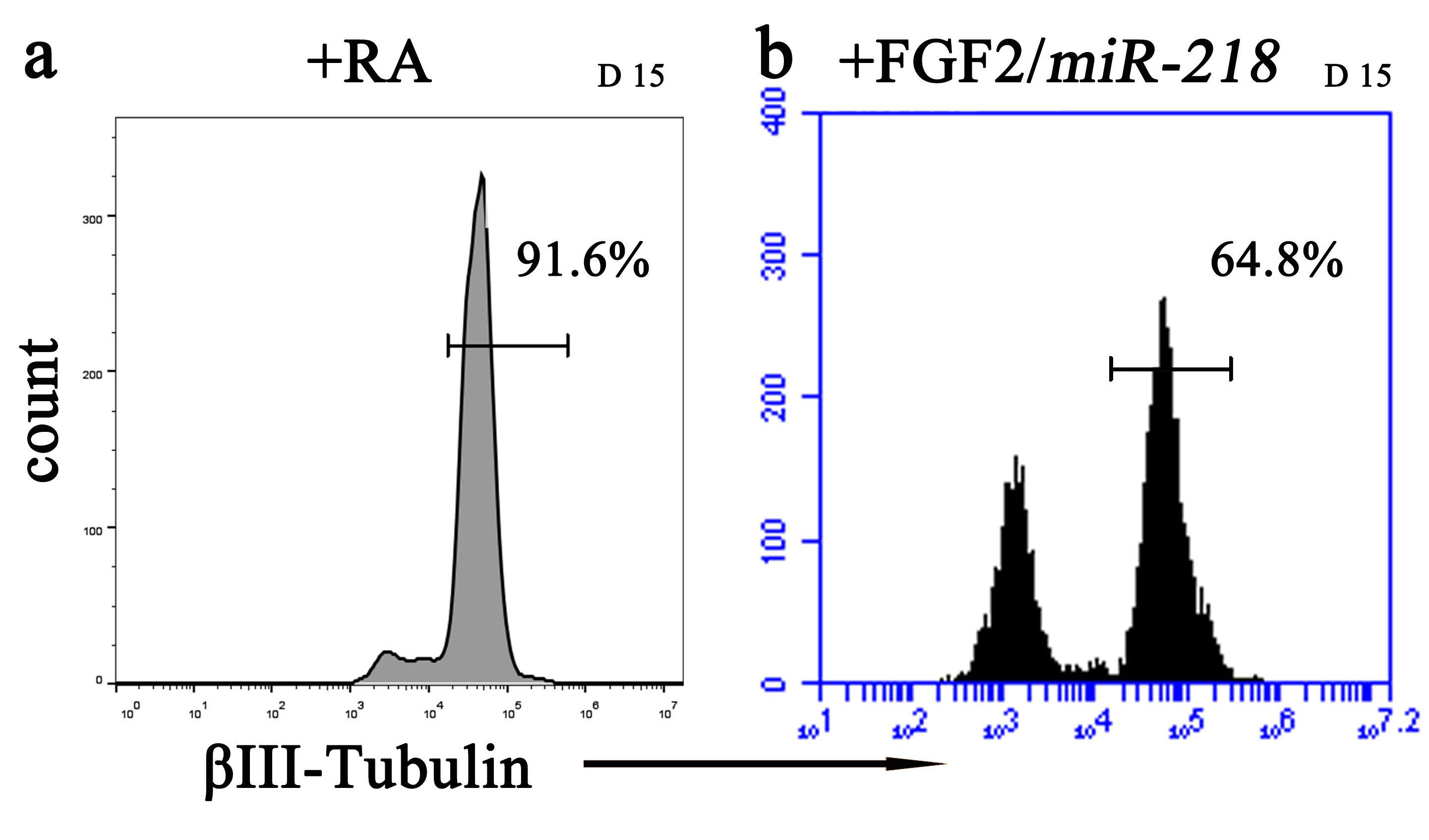


Supplementary Fig. S 2

After the cells are incubated with RA at 5 time points (Day 0, Day 2, Day 5, Day 10 and Day 15), the percentage of ASCs neuronal differentiation are estimated by flow cytometric analysis (n=2).


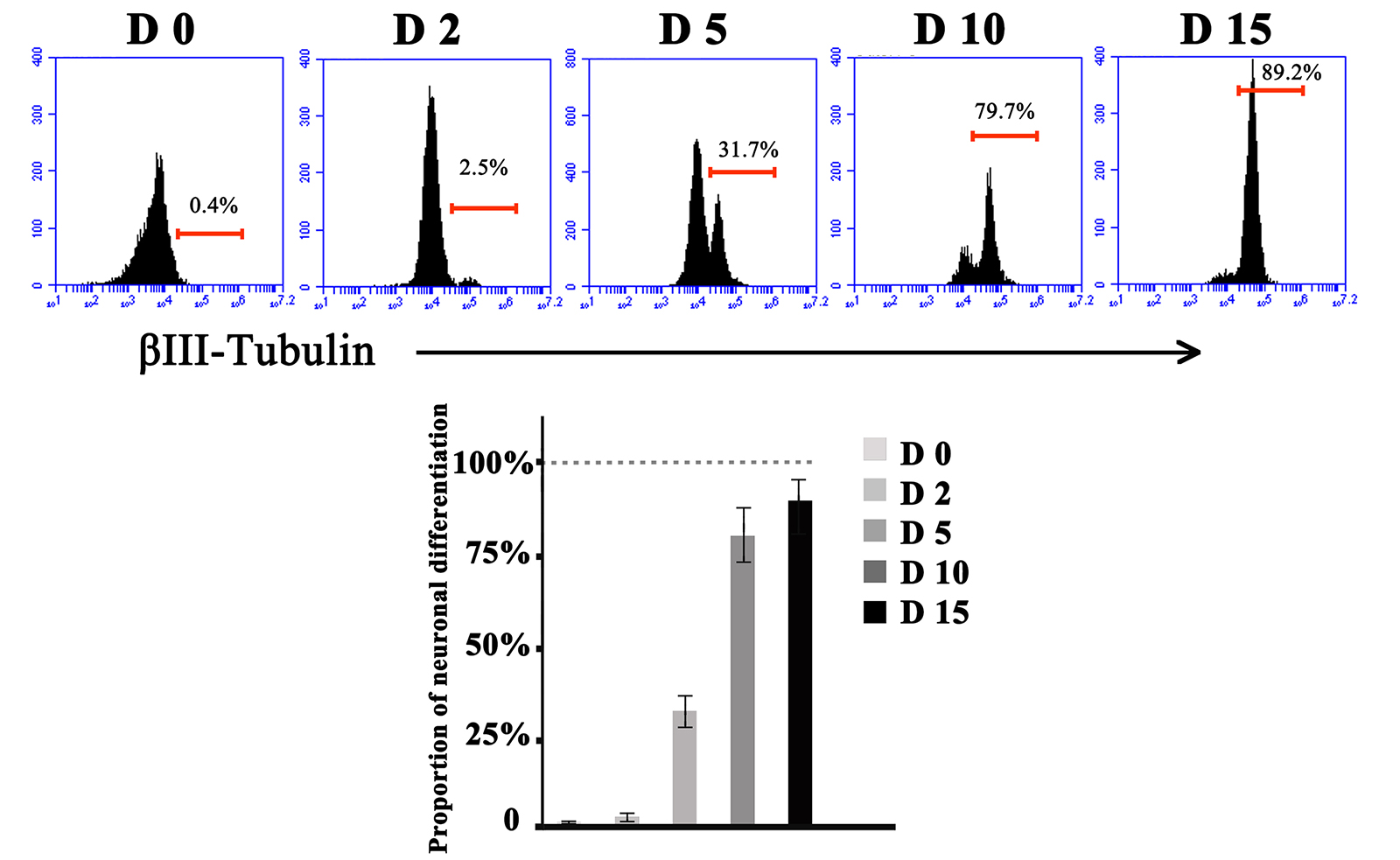


Supplementary Fig. S 3

The expression levels of *miR-9*, *miR-146a* and *miR-214* are monitored at three time points (Day 0, Day 5 and Day 15) by qRT-PCR (* P < 0.05, ** p < 0.01, n=3).


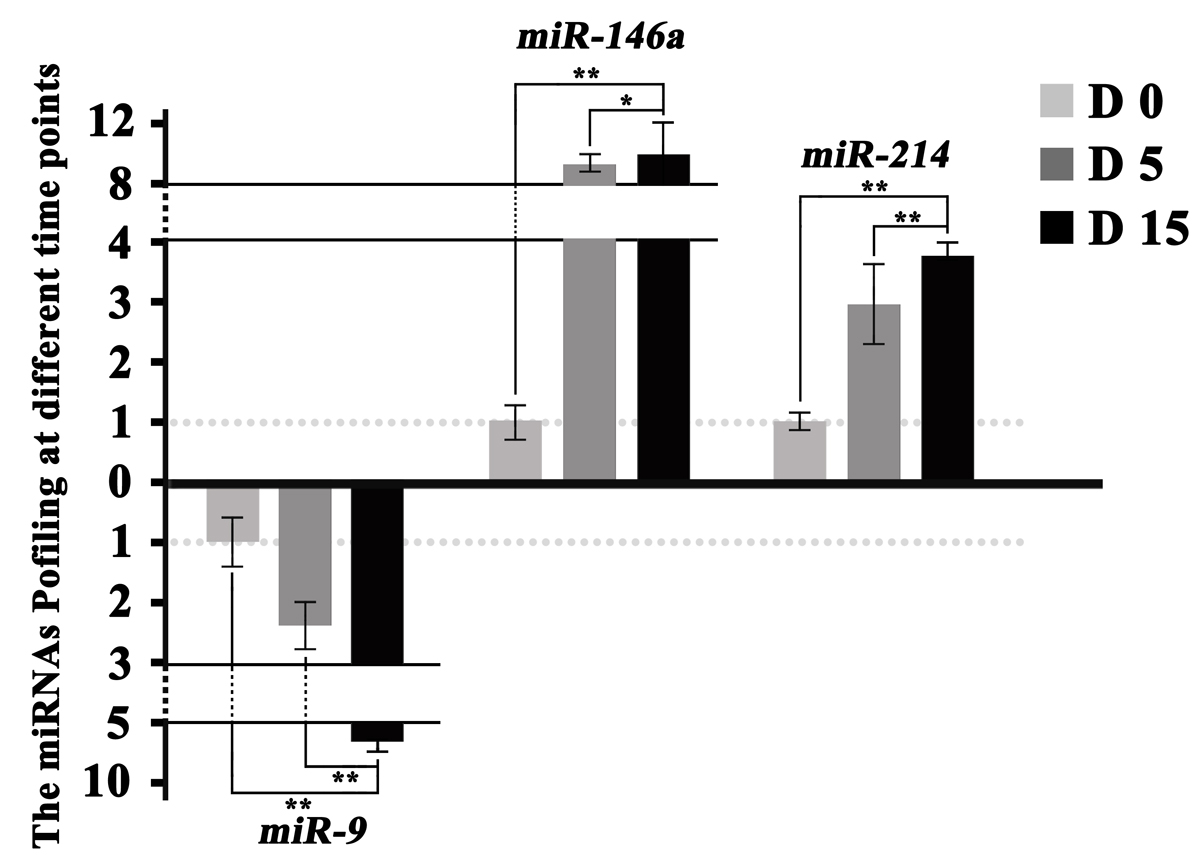


Supplementary Fig. S 4

The relative *miR-218* expression levels of control group and anti-Wnt group (the medium is added with ICG-001 protein for 2 days, D 2) are detected by qRT-PCR (n=2).


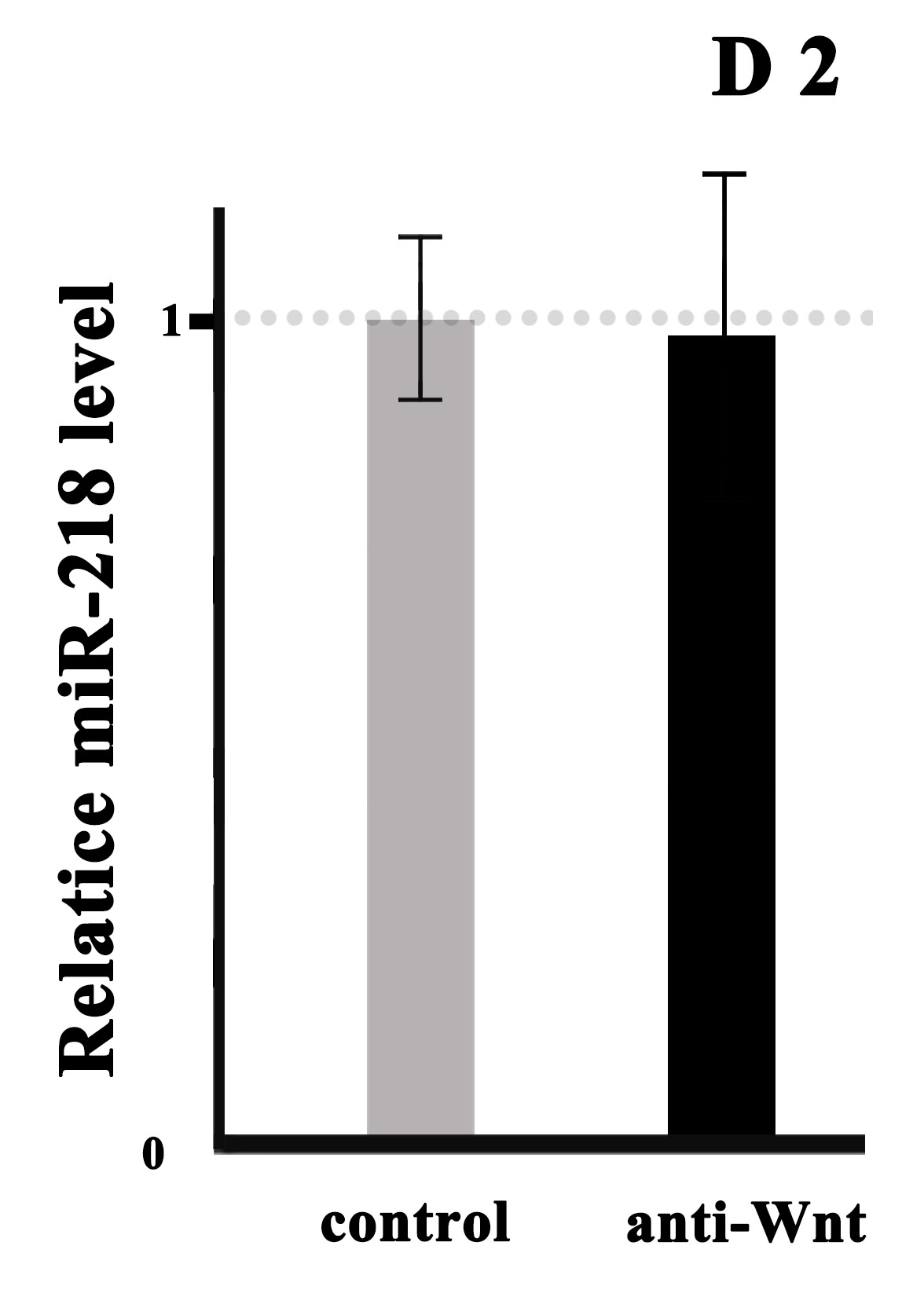


Supplementary Fig. S 5

The feedback loop between FGF2 and *miR-218* is confirmed by the qRT-PCR. (a) The endogenous *Fgf2* mRNA expression levels in control (without any treated, -FGF2/ -*miR-218* group), -FGF2/ miR-NC, -FGF2/ anti-*miR-218* and -FGF2/ *miR-218* group are monitored by the qRT-PCR at the Day 2 (the miRNAs plasmids were transfected cell for 48h, n=4). (b) The relative *miR-218* expression levels in control (without any treated, -FGF2/ -*miR-218* group), +FGF2, +FGF2/ miR-NC, +FGF2/ anti-*miR-218* and +FGF2/ *mi**R-218* group are detected by the qRT-PCR at the Day 12 (the FGF2 was added to the medium and maintain for 10 days and transfect the plasmids for 48h, n=3, **p < 0.01).


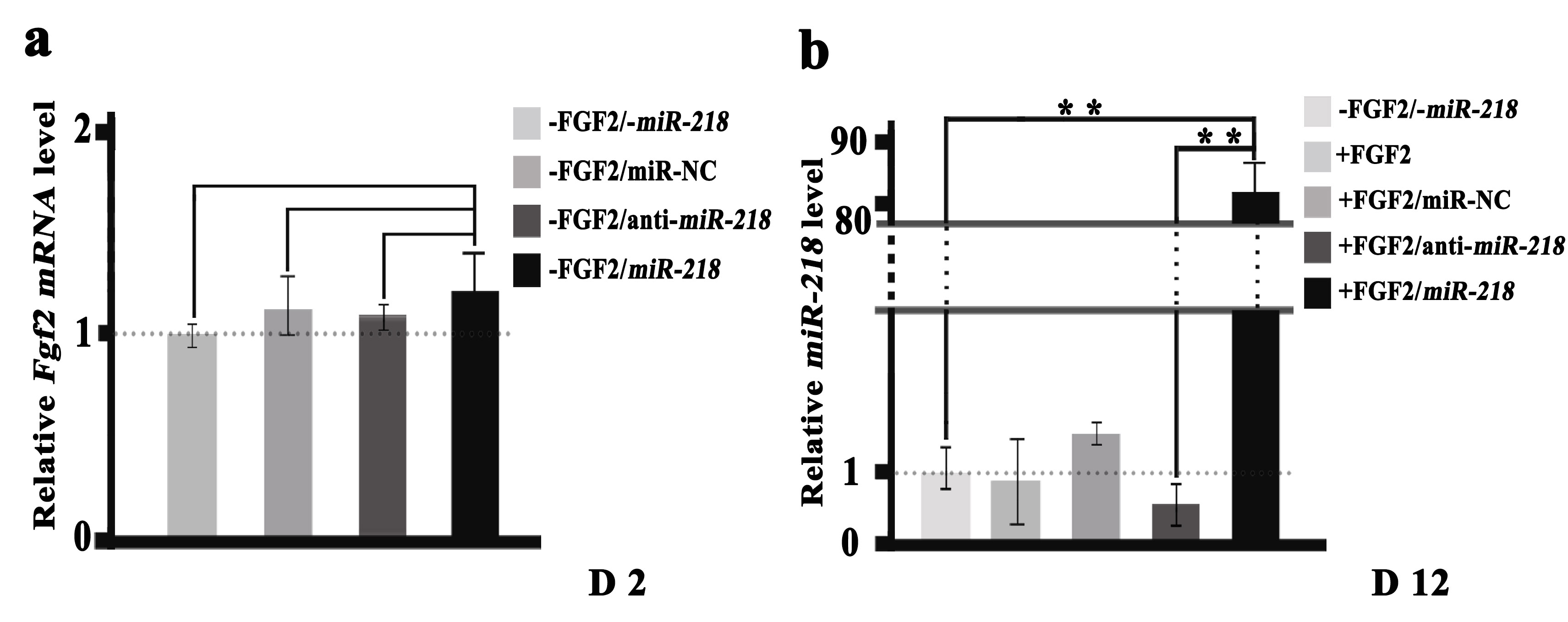


Supplementary Fig. S 6

The Western blotting analysis of FGF signaling marker proteins expression when the ASCs medium was added 10ng/mL FGF2. After 10 days, the cells from the groups of FGF2 (+FGF) and control (-FGF2) were extracted and the FGF signaling markers expression (ERK1/2, Santa Cruz, Catalog: SC-81492; p-ERK1/2, Immunoway, Catalog: YM3518; PLCγ1, Santa Cruz, Catalog: SC-81; p-PLCγ1, Santa Cruz, Catalog: SC-12943) were detected in the –FGF2 and +FGF2 groups (** p < 0.01, n=2).


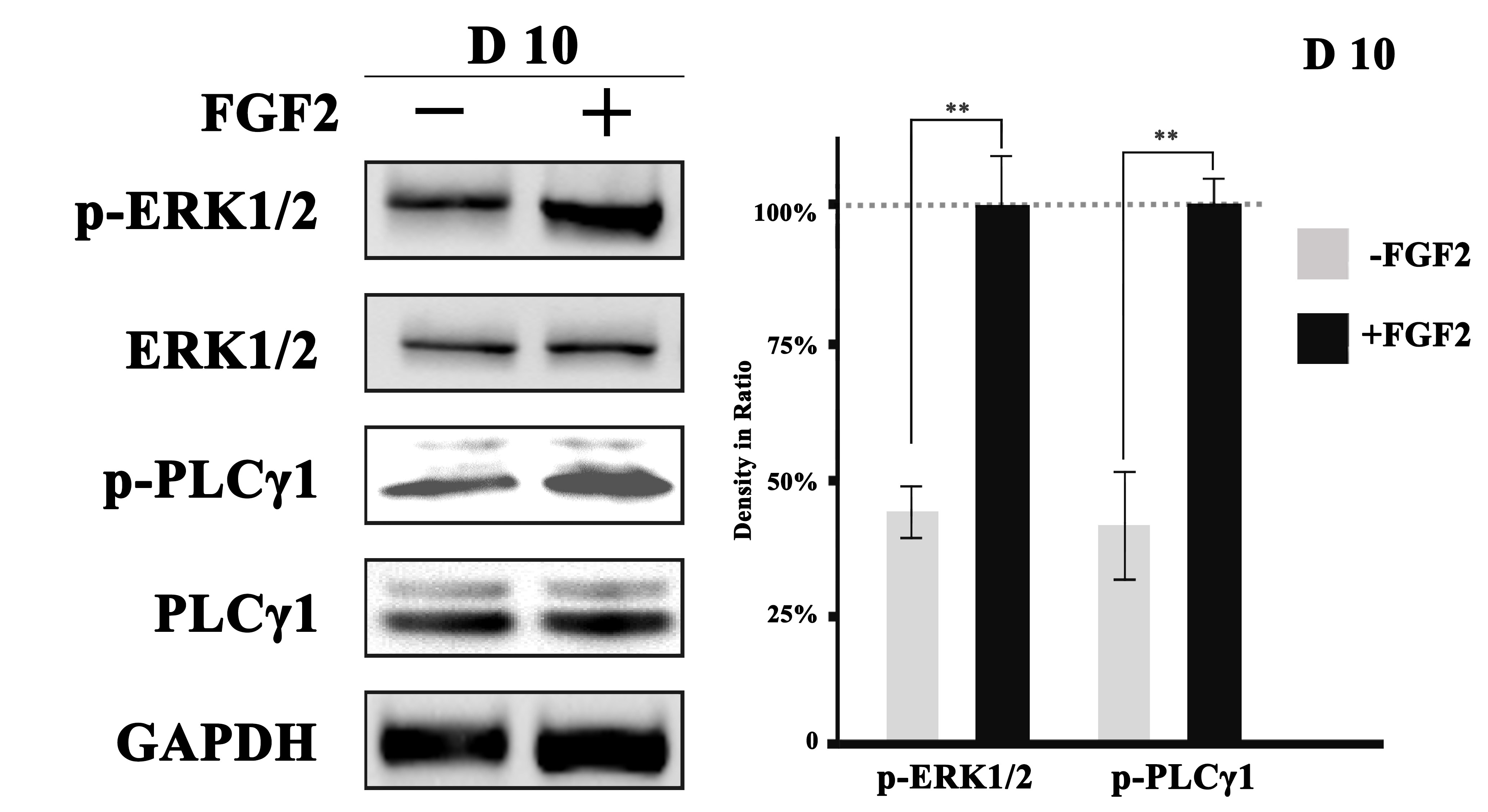

Supplement: Supplementary Dataset [file srep39427-s1.doc]
